# Technical efficiency of rural primary health care system for diabetes treatment in Iran: a stochastic frontier analysis

**DOI:** 10.1186/s40200-017-0312-8

**Published:** 2017-08-14

**Authors:** Mostafa Qorbani, Farshad Farzadfar, Reza Majdzadeh, Kazem Mohammad, Abbas Motevalian

**Affiliations:** 1 Department of Epidemiology, School of Public Health, Iran University of Medical Sciences, Tehran, Iran; 2 Non-communicable Diseases Research Center, Alborz University of Medical Sciences, Karaj, Iran; 3 Non-communicable Diseases Research Center, Endocrinology and Metabolism Population Sciences Institute, Tehran University of Medical Sciences, Tehran, Iran; 4 Department of Epidemiology and Biostatistics, School of Public Health, Tehran University of Medical Sciences, Tehran, Iran

**Keywords:** Stochastic frontier analysis, Technical efficiency, Human resources, Diabetes mellitus

## Abstract

**Background:**

Our aim was to explore the technical efficiency (TE) of the Iranian rural primary healthcare (PHC) system for diabetes treatment coverage rate using the stochastic frontier analysis (SFA) as well as to examine the strength and significance of the effect of human resources density on diabetes treatment.

**Methods:**

In the SFA model diabetes treatment coverage rate, as a output, is a function of health system inputs (Behvarz worker density, physician density, and rural health center density) and non-health system inputs (urbanization rate, median age of population, and wealth index) as a set of covariates. Data about the rate of self-reported diabetes treatment coverage was obtained from the Non-Communicable Disease Surveillance Survey, data about health system inputs were collected from the health census database and data about non-health system inputs were collected from the census data and household survey.

**Results:**

In 2008, rate of diabetes treatment coverage was 67% (95% CI: 63%–71%) nationally, and at the provincial level it varied from 44% to 81%. The TE score at the national level was 87.84%, with considerable variation across provinces (from 59.65% to 98.28%).Among health system and non-health system inputs, only the Behvarz density (per 1000 population)was significantly associated with diabetes treatment coverage (β (95%CI): 0.50 (0.29–0.70),*p* < 0.001).

**Conclusion:**

Our findings show that although the rural PHC system can considered efficient in diabetes treatment at the national level, a wide variation exists in TE at the provincial level. Because the only variable that is predictor of TE is the Behvarz density, the PHC system may extend the diabetes treatment coverage by using this group of health care workers.

## Background

Although the number of people living with and dying of diabetes mellitus (DM) is increasing across the world [1, 2] the major increase is occurring in low and middle-income countries [3]. It has been reported that the prevalence rate of type 2 diabetes in Iran among 25 to 64 year-old age group is 11.37% (95%CI: 9.86–12.89) [4]. According to a recently conducted study in Iran, DM treatment coverage was 39.2% in 2005 [5]. In Iran, the National Plan for Diabetes Control and Prevention (NPDCP) was developed and implemented between 1996 and 2002,in order to prevent and control DM and its complications in an early phase through screening ‘individuals at risk’ and pregnant women. The NPDCP is integrated into the primary health system (PHC) of Iran [6]. As a part of its protocol in rural areas, Iranian rural primary health workers (Behvarzes) are trained to identify high-risk groups and refer them to physicians in health houses for DM testing and, if required, treatment. The main duties of Behvarz are taking care and follow up of patients.^6^

Human resources for health are known to have imbalanced distribution in low and middle income countries [7] and a few studies have investigated the association between this geographical imbalance in the distribution of human resources for healthand health outputs or intervention coverage [8–16]; the results of these studies were contradictory.

The first study that measured the efficiency of health systems in attaining health outputs applying production function models was the world health report in 2000 [17]. Technical efficiency (TE) was defined as the ability to produce the maximum possible output from a given set of inputs and wasmeasured in terms of the relationship between actual level of health output and the maximum attainable output for the observed inputs [18].Stochastic frontier analysis (SFA) is a parametric technique for estimating efficiency in health care systems, which is has been introduced and used recently.The SFA uses regression analysis to estimate the production frontier and measures the efficiency of a unit using the residuals from the estimated equation. In this method, in addition to a random error, another error term is included which represents systematic inefficiency.

In this study, we explored the TE of Iranian rural PHC system for DM treatment coverage rate using the SFA technique in 2008. We also tested the strength and significance of the effect of health and non-health inputs on levels of DM treatment coverage.

## Methods

### Study area and data set

In this cross-sectional study, rural areas of all provinces in Iran in 2008 were assessed for estimating TE of the rate of self-reported DM treatment coverage using the SFA model. According to this model DM treatment coverage rate as the output was a function of health system inputs (such as Behvarz worker density, physician density and rural health house density)and non-health system inputs (urbanization rate, median age of population, and wealth index) as a set of covariates. The data about the rate of DM treatment coverage was obtained from the Non-Communicable Disease Surveillance Survey (NCDSS) 2008; the data about health human resources and rural health houses density were collected from the health census database called D-Tarh 2008; median age, and rate of urbanization were collected from the census datas 2006 and 2011; and wealth index were collected from the household survey of 2008.

### Definitions

The rate of self-reported DM treatment coverage was defined as the proportion of people with diabetes who reported taking medication for diabetes treatment. Rural health center, Behvarz, and physician density are defined as the ratio of the number of rural health centers, Behvarzes and physicians per 1000 total populations. Median age of population was estimated by interpolating population characteristics attained by census 2006 and 2011 using the Growth Method. Urbanization rate was defined as the ratio of urban to rural population which was estimated by interpolating population characteristics attained by census 2006 and 2011 years using Growth method. According to previous evidence in the “Progress in the International Reading Literacy Study” (PIRLS) [19] and by using principle component analysis (PCA) method, family assets and characteristics of living place were summarized in one main variable to construct wealth index. This main variable explained 69% of total variance.

### Conceptual framework of SFA model

In present study we used the SFA model suggested by Battese and Coelli [20]. The model is as follows:

$$ yi={X}_i\beta +{v}_i-{u}_i $$

Where y_i_ is the logarithm of the output in province *i*, *X*_*i*_ is the vector of inputs associated with province *i*, β is the vector of parameters to be estimated, *v*_*i*_ is the random error components and *u*_*i*_ is the inefficiency component. The noise component v_i_, have a normal distribution N(0,σ^3^_u_) and u_i_ which are nonnegative random variables, have truncated normal distribution; N^+^ (μ,σ^3^_u_) . The μ of the truncated normal distribution is modeled as a linear function of a set of covariates.

According to this SFA model, we modeled DM treatment coverage *(C*_*i*_ *)* as a function of a vector of inputs *x*_*i*_ for the *i* ^*th*^ province, *C*_*i*_ *= f(x*_*i*_ *)*. The x_i_ vector of inputs is composed of Behvarz density *B*_*i*_, physician density *P*_*i*,_ rural health center density *RHC*_*i*_,

$$ {TE}_i={C}_i/f\ {\left({B}_i,{P}_i,{RHC}_i;\beta \right)}^{\ast}\mathit{\exp}.\left({v}_i\right) $$

The stochastic frontier model *(f(B*_*i*_ *, P*_*i*_ *, RHC*_*i*_ ; β)* *exp.(v*_*i*_ *))* consists of two parts: a deterministic part and a producer-specific part (exp(v_i_)) which captures the effect of random variables on each producer. Deterministic part [*f (B*_*i*_ *, P*_*i*_ *, RHC*_*i*_ *; β]* takes the log-linear Cobb–Douglas form^24^:

According to above formula, TE_i_ = 1 if observed output *(C*_*i*_ *)* is equal to maximum attainable output in an environmental characterized by *exp.(v*_*i*_ *)*. TE_i_ < 1 when observed output *(C*_*i*_ *)* is less than maximum attainable output in an environmental characterized by *exp. (v*_*i*_ *)* which can be vary across provinces.

## Results

In 2008, the rate of DM treatment coverage in rural areas was 67% (95% CI: 63 to 71), nationally. In this year, the density of Behvarz workers was 1.61 per 1000 total population (with range of 0.84 to 2.30) and the density of physicians per 1000 total populations was 0.29 (range: 0.18–0.52) (Table 1).

**Table 1:** Descriptive statistics of input and output variables in the SFA model for DM treatment coverage in rural areas of Iran, 2008

Variable	Mean	95%CI	Minimum	Maximum
DM treatment coverage (self-report)	0.67	0.63–0.71	0.44	0.81
Physician density^a^	0.29	0.26–0.32	0.18	0.52
Behvarz density^a^	1.61	1.5–1.7	0.84	2.30
Rural health center density^a^	0.13	0.11–0.14	0.08	0.19
Wealth index	0.004	−0.35-0.35	−1.96	1.66
Urbanization rate	0.66	0.6–0.7	0.50	0.95
Median age	24.5	23.8–25.3	19.6	28

^a^Per 1000 total population

**Fig. 1:**
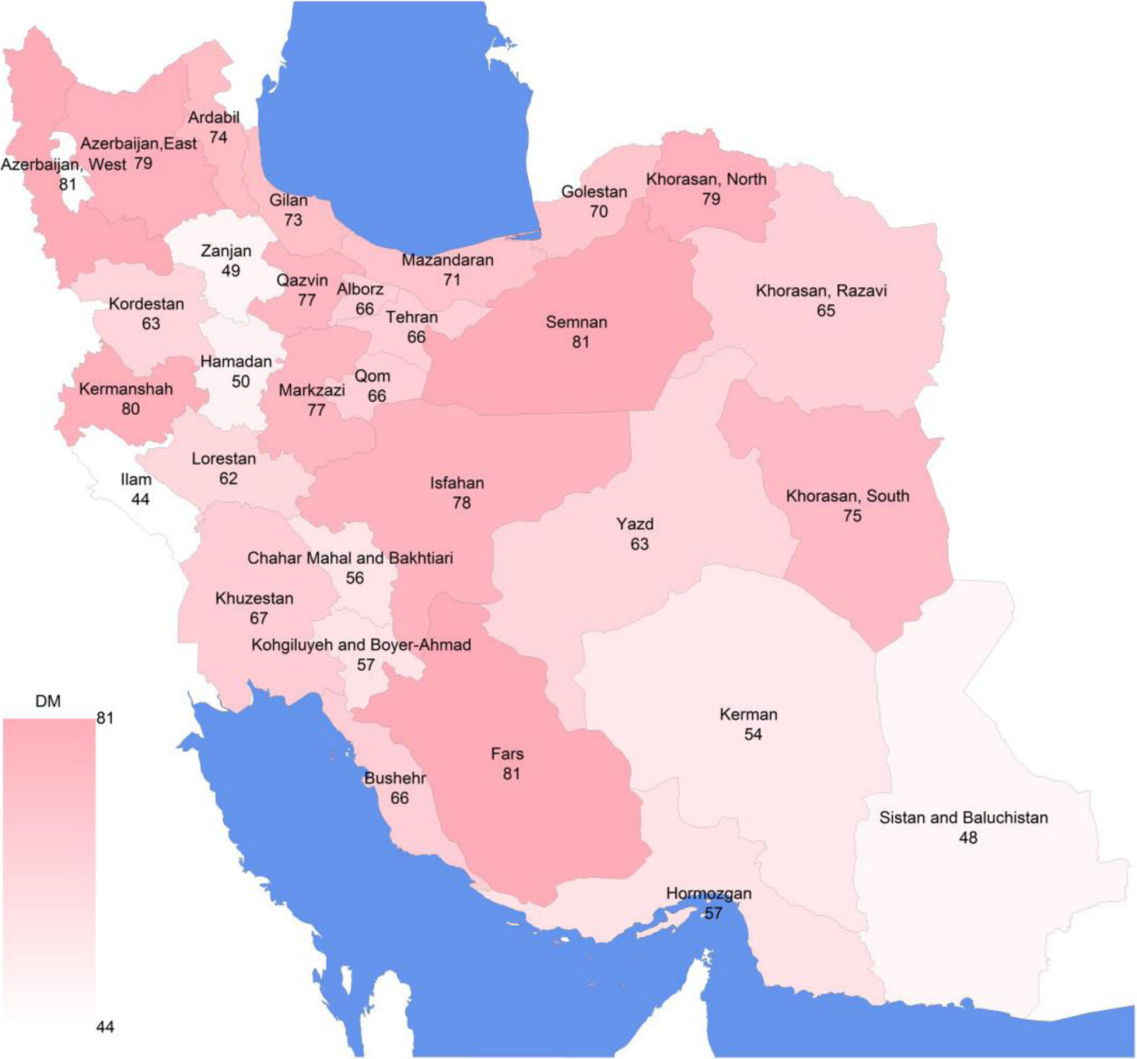
DM treatment coveragein rural areas of Iran at provincial level, 2008

Figure 1 shows the rate of DM treatment coverage at the provincial level in Iran, 2008. This rate varies considerably across provinces from 44% (Ilam province) to 81% (West Azerbaijan province).

Table 2 presents the results of the SFA model for TE of the PHC system for DM treatment coverage rate. Only the effect of Behvarz worker density (per 1000 total population) on DM treatment coverage rate was statistically significant (β coefficient: 0.50, 95%CI: 0.29–0.70), whereas the effect of physician density on this rate was not statistically significant (β coefficient: 0.05, 95%CI: -0.12-0.22).The results of Wald *x* ^3^ statistics showed that the PHC system was not efficient in attaining DM treatment coverage rate (Wald *x* ^3^: 30.35, *p*-value < 0.001). In addition, the estimate for variance parameter of inefficiency components (σ^3^_*u*_)indicated that this effects was statistically significant (*P*-value < 0.001).

**Table 2:** Coefficients of the SFA model for DM treatment coveragein rural areas of Iran, 2008

Independent variable	Coefficients	95%CI	*P*-value
Production function			
Behvarz density (per 1000 total population)	0.50	0.29, 0.70	<0.001
Physician density (per 1000 total population)	0.05	−0.12,0.22	0.60
Rural health center density (per 1000 total population)	−0.07	−0.08,0.21	0.38
Wealth Index	−0.51	−7.2, 6.23	0.88
Urbanization rate	−2.86	−15.58,9.86	0.65
Median age (years)	−17.27	−43.86,9.32	0.20
Constant	−0.47	−0.74,-0.19	0.001
Distribution of u and v			
σ^3^_u_	0.15	0.11,0.20	<0.001
σ^3^_v_	0.06	0.03,0.08	0.35
Number of observation: 31			
Wald x^3^	30.35		<0.001

**Fig. 2:**
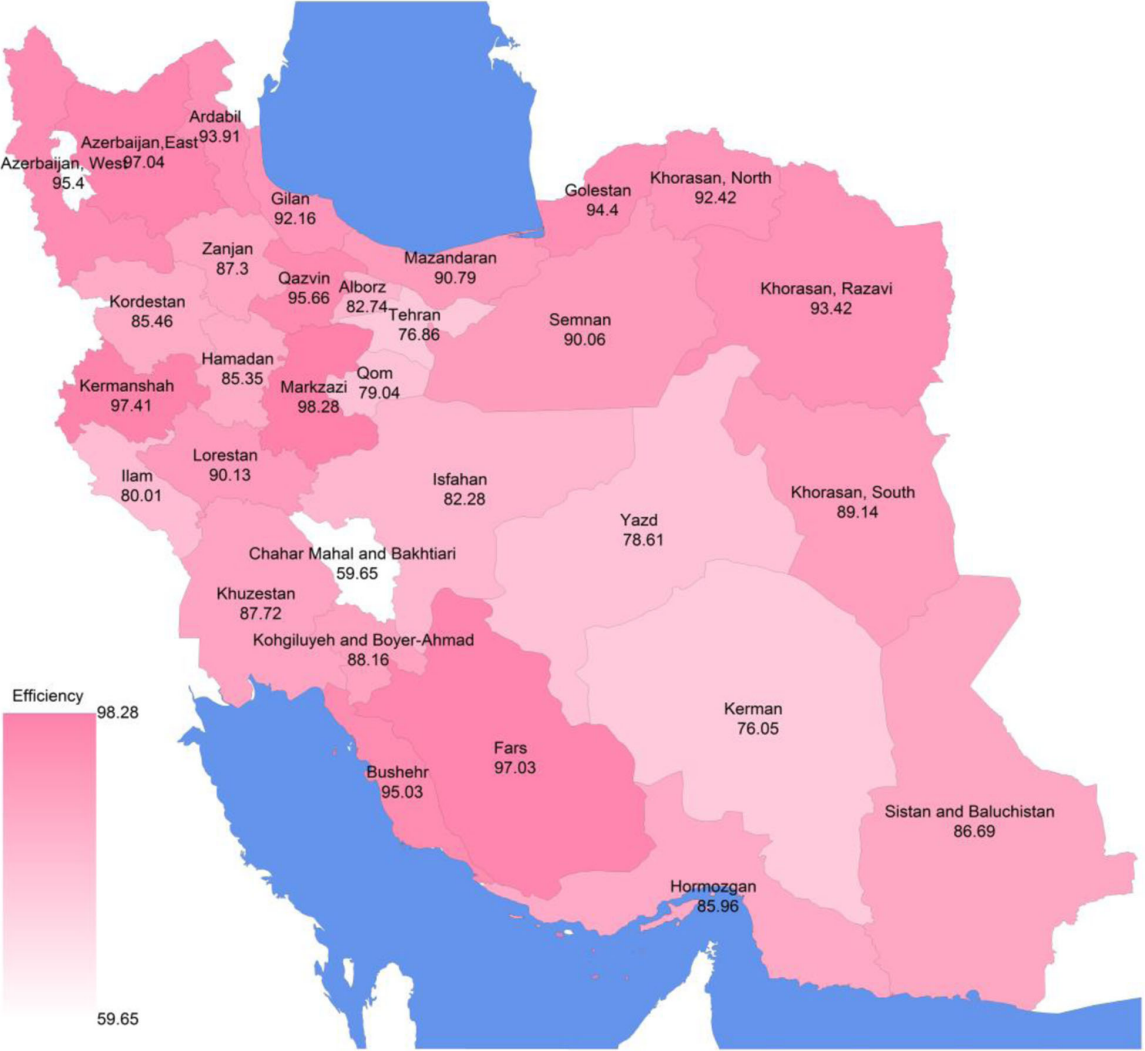
Technical efficiency of rural primary health care system for DM treatment coverage at provincial level in Iran, 2008

Table 3 shows the estimated TE for the PHC system at the provincial level, using the SFA model. The TE score at the national level was 87.84%, with considerable variation across provinces (from 59.65% (Chaharmahal & Bakhtiari) to 98.28% (Markazi province)). Figure 2 demonstrates TE of Iranian PHC system for DM treatment at provincial level.

**Table 3:** DM treatment coverage and technical efficiency at national and provincial level in rural areas of Iran, 2008

ID	Province	Number of rural total population	DM treatment coverage%	Efficiency %
1	Markazi	408,630	77	98.28
2	Kermanshah	616,599	80	97.41
3	East Azerbaijan	410,522	79	97.04
4	Fars	1,578,334	81	97.03
5	Qazvin	297,505	77	95.66
6	West Azerbaijan	1,072,773	81	95.40
7	Boshehr	280,057	66	95.03
8	Golestan	833,255	70	94.40
9	RazaviKhorasan	1,752,463	65	93.42
10	Ardabil	477,219	74	93.91
11	North Khorasan	382,365	79	92.42
12	Gilan	1,044,515	73	92.16
13	Mazandaran	1,311,380	71	90.79
14	Lorestan	672,328	62	90.13
15	Semnan	120,825	81	90.06
16	South Khorasan	246,589	75	89.14
17	Kohgiluyeh&Boyer-Ahmad	350,286	57	88.16
18	Khozestan	1,229,588	67	87.72
19	Zanjan	395,989	49	87.30
20	Sistan and Baluchestan	942,249	48	86.69
21	Hormozgan	663,401	57	85.96
22	Kurdestan	611,689	63	85.46
23	Hamadan	724,877	50	85.35
24	Alborz	130,883	66	82.74
25	Isfahan	622,441	78	82.28
26	Ilam	209,650	44	80.01
27	Qom	48,870	66	79.04
28	Yazd	184,674	63	78.61
29	Tehran	395,202	66	76.86
30	Kerman	895,607	54	76.05
31	Chaharmahal&Bakhtiari	1,087,876	56	59.65
-----	National	19,998,643	67	87.84

## Discussion

According to our results, nationally DM treatment coverage rate in rural areas was 67%(95% CI: 63 to 71). Farzadfar et al. reported that DM treatment coverage rate in urban and rural areas in 2005 according to the NCDSS was 39.2% (95% CI 37·7 to 40·7); this discrepancy can be attributed to the definition of DM treatment coverage; in our study, DM treatment coverage rate was based on self-report which may justify the observed higher rate [5].

We also found a significant association between Behvarz worker density and DM treatment coverage rate. Our results show that each additional Behvarz worker density per 1000 total population increased DM treatment coverage rate by 0.5(95% CI: 29–70), consistent with Farzadfar et al. who show that each additional Behvarz worker density per 1000 total population decreases fasting plasma glucose by 0.09 mmol/L (0.01 to 0.18) on average [5]. Findings from other studies on the association between the density of human resources and health system outputs are contradictory. Some show that the density of health-care workers is associated with improved health system outputs [8–11]; however other do not support this association [12–16]. Most studies have conducted cross-country analyses, but the associations at subnational level are considered by only a few of them [5]. Previous studies showed that in developing countries efficient utilization of human resources for attaining health outputs at the subnational units faces two major problems: uneven distribution of health workers at the subnational level, and difficulty in identifying the threshold of human resource density to achieve the minimum acceptable level of health outputs [7–22]. To address these problems the WHO in 2006 dedicated world health reports to the topic of human resources for attaining health outputs, and declared that “health workers are crucial for attaining acceptable heath outputs through the PHC system” and that “achieving PHC and Millennium Development Goals are possible only by an appropriately prepared and efficient health workers” [23].

In present study, the effect of physician density (unlike that for Behvarz density) was not statistically significant on DM treatment coverage, in line with Farzadfar et al.’s study [5]. The likely reason for this observation has to do with the defined roles of these groups in the Iran health system. Iran has a National Diabetes Control and Prevention Program, with specific roles for Behvarz workers. Thus, although physicians in the PHC system also have a key role in the definite diagnosis and treatment of referred patients in rural areas, Behvarz workers are in charge of the initial identification of patients according to a determined protocol as well as supervision of their diet and life style modifications, along with follow- ups to check that patients take their medication and visit physicians periodically, if they have symptoms of DM.

We found a wide variation in the levels of TE across Iranian provinces, from 59.65% to 98.28% which suggests that some of provinces could increase their level of DM treatment coverage by using their human resources, particularly Behvarz workers, more efficiently. Overall, the average score of efficiency was 87.84(%). This implies that the provinces, in terms of TE, could produce the same level of DM treatment coverage by using 12.16(%) fewer resources (inputs).

Previous studies have shown that increasing the number of human resources for health, especially Behvarz worker, with well-defined guidelines is a major strategy for attaining acceptable management of NCD [5]. However; our finding suggests that the PHC may extend the coverage of DM treatment by using Behvarz worker more efficiently. In other words, on average, provinces are using more inputs than they need.

To the best of our knowledge, the present study is the first study in Iran that estimates TE of human resources for attaining DM treatment coverage at the sub-national level using the SFA model.

There were some limitations in our study. The main one was the definition of DM treatment coverage which was based on self-report. In addition using survey data for calculating output instead of obtaining data from rural health house may limits our data. We also were not able to include public health expenditure per capita of each province in the SFA model.

## Conclusion

Our findings show that although the rural PHC system can be considered efficient in DM treatment coverage at the national level; a wide variation exists in TE at the provincial level. Because the only variable that is predictor of TE is the Behvarz density, the PHC system may extend DM treatment coverage by using this group of health care workers. Our results can help health policy makers to attain higher health outputs by using community health workers; we recommend that the PHC system in Iran rely more on this type of workforce, which perhaps is more cost effective than employing physicians.

### Endnote

Any substance to which subjects were sensitive were mentioned it in the questionnaire.

## Abbreviations

DM: Diabetes mellitus
PHC: Primary health care
SFA: Stochastic frontier analysis
TE: Technical efficiency.
